# Effect of fuchsin fixation of pollen on DNA barcode recovery

**DOI:** 10.1002/ece3.10475

**Published:** 2023-08-31

**Authors:** Melanie B. Streicher, Steven D. Johnson, Sandi Willows‐Munro

**Affiliations:** ^1^ Centre for Functional Biodiversity, School of Life Sciences University of KwaZulu‐Natal Scottsville South Africa; ^2^ School of Life Sciences University of KwaZulu‐Natal Scottsville South Africa

**Keywords:** barcoding, fuchsin, ITS, pollen identification

## Abstract

Pollen grains attached to insects are a valuable source of ecological information which can be used to reconstruct visitation networks. Morphological pollen identification relies on light microscopy with pollen usually stained and mounted in fuchsin jelly, which is also used to remove pollen from the bodies of insects. Pollen embedded in fuchsin jelly could potentially be used for DNA barcoding and metabarcoding (large‐scale taxonomic identification of complex mixed samples) and thus provide additional information for pollination networks. In this study, we determine whether fuchsin‐embedded pollen can be used for downstream molecular applications. We evaluate the quality of plant barcode (ITS) sequences amplified from DNA extracted from both fresh (untreated) pollen, and pollen which had been embedded in fuchsin jelly. We show that the addition of fuchsin to DNA extraction does not impact DNA barcode sequence quality during short‐term storage. DNA extractions from both untreated and fuchsin‐treated pollen produced reliable barcode sequences of high quality. Our findings suggest that pollen which has been collected, stained, and embedded in fuchsin jelly for preliminary microscopy work can be used within several days for downstream genetic analysis, though the quality of DNA from pollen stored in fuchsin jelly for extended periods is yet to be established.

## Graphical abstract

In this study we determine whether fuchsin‐embedded pollen can be used for downstream molecular applications. We evaluate the quality of plant barcode (ITS) sequences amplified from DNA extracted from both fresh (untreated) pollen, and pollen which had been embedded in fuchsin jelly. Our study demonstrates that good quality sequence data can be obtained from fuchsin embedded pollen. This will allow visitation studies to include data from both morphological and molecular techniques. Understanding plant‐pollinator interactions has implications for the study of ecosystem evolution and given that pollination is key ecosystem service the conclusions of this manuscript also have important socio‐economic impacts.

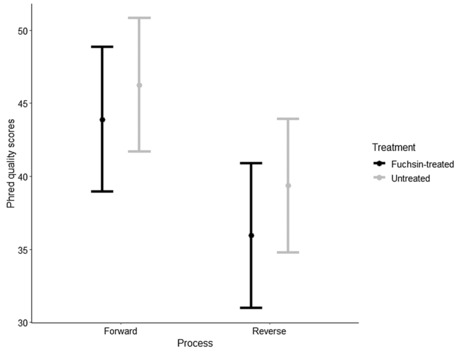

## 1 INTRODUCTION

Pollination networks were traditionally studied and constructed by direct observation of interactions (Dupont et al., 2009; King et al., 2013) or by tracking the transferred pollen load using palynological techniques (Arceo‐Gómez et al., 2020; Wood & Roberts, 2018). The latter methodology is time‐consuming and relies on well‐curated and comprehensive pollen reference libraries (Elliott & Jonathan Davies, 2014; Kraaijeveld et al., 2015). The scale of such research is thus often limited. Although the input costs are presently still high, DNA barcoding and metabarcoding is currently an emerging method for rapid species identification involving plant–pollinator interactions (Arstingstall et al., 2021; Keller et al., 2015).

The success of DNA‐based methods depends on many factors including the gathering, processing, and storing of specimens (Dillon et al., 1996; Willows‐Munro & Schoeman, 2015; Zimmermann et al., 2008). Metabarcoding methods are also sensitive to contamination and have poor quantitative power (Lamb et al., 2019); the taxa amplified depend on primer specificity, data may include sequencing errors, and data may incur false positives or negatives (Bell et al., 2017; Cuff et al., 2022). It is therefore desirable to use a combination of traditional morphology‐based and newer molecular‐based methods in order to characterise network structural properties (Bosch et al., 2009; Pornon et al., 2017).

Barcoding and metabarcoding have the advantage of discriminating pollen of closely related species, which is seldom possible using light microscopy alone (Sickel et al., 2015). Several barcode primers can be ‘pooled’ to amplify an array of target DNA (e.g. allowing for simultaneous barcoding of pollinator and pollen) at a comparatively minimal increase in cost. It is, however, possible for ‘rare’ taxa to be underrepresented in molecular data, depending on the chosen markers and quantity of pollen grains (Bell, Burgess, et al., 2016). It is also possible for errors to be incorporated during DNA replication which, once amplified, could significantly distort ecological interpretations (Bandelt et al., 2001; Kanagawa, 2003). False positives and negatives are a concern too (Farrell et al., 2021). These inaccuracies are especially problematic in forensic fields (Bandelt et al., 2001). For ecological studies in regions where a comprehensive barcode reference library linking DNA sequence data to taxonomically identified specimens exists, potential contamination, misidentifications, and other sequencing errors can be ruled out. Additionally, both field and laboratory practices and protocols need to be standardised to eliminate errors arising from inter‐personal handling techniques (Farrell et al., 2021; Raclariu et al., 2018) and to allow for the integration of results from different studies. In the case of pollen, DNA extraction methods are destructive, with original samples not available for cross‐referencing at a later stage (Bell, Burgess, et al., 2016).

When using traditional microscopy methods, the integrity of the pollinator and pollen remains intact for museum storage and future palynological analyses. Analysing the pollen loads by light microscopy and precise, targeted swabbing, allows one to make inferences about specialisation and pollen placement (Walton et al., 2020). This typically involves the use of dye prior to being viewed by light microscopy (Beattie, 1971; Jia et al., 2021; Wodehouse, 1929). Fixing pollen to slides with fuchsin jelly (Beattie, 1971) has become a routine and popular protocol for removing pollen from insects and then visualising pollen exine morphology (Jia et al., 2021). Preparing semi‐permanent fuchsin pollen mounts is a cost‐effective and non‐pathogenic for human use when phenol is eliminated (Umroong, 2021). Fuchsin, when used as an exine‐specific dye for staining, attaches to the pollen coat which comprises sporopollenin—a chemically inert and robust biopolymer (Jia et al., 2021; Mackenzie et al., 2015). Although soluble in strong oxidising agents which renders stored DNA susceptible to damage, the pollen coat does not disintegrate when in contact with other organic and inorganic acids and bases (Southworth, 1974).

For molecular approaches to studying natural systems, methods of gathering and storing biological material can influence the success of downstream analyses (Dillon et al., 1996). Both the quantity and quality of extracted DNA and thus the PCR efficiency can be decreased by substandard methods of collection and storage. Early‐stage PCR misincorporations, resulting in less reliable final sequences, are more likely to occur when template DNA is of poor quality (low concentration, possible contamination, damaged/degraded DNA; Casbon et al., 2011; Dillon et al., 1996). Contrary to previous assumptions, killing method (ethyl acetate, cyanide and freezing) and post‐mortem storage of insect specimens has been shown not to hazard downstream DNA recovery for insect taxa (Willows‐Munro & Schoeman, 2015). This raises the possibility that pollen collected and embedded in fuchsin jelly may also be suitable for DNA analysis. However, pollen embedded in fuchsin jelly has not previously been considered for downstream molecular applications, nor has it been tested.

Fuchsin jelly contains several ingredients: glycerine; gelatine; crystalline basic fuchsin stain, and sometimes crystalline phenol, which may have an effect on genetic material (Massie & Zimm, 1965) and may inhibit downstream PCR‐based amplification. Fuchsin staining has been shown to inhibit PCR DNA amplification only minimally in histological samples (Murase et al., 2000). However, the heating, melting and setting of the fuchsin jelly may also affect the stability of the pollen‐derived DNA.

Depending on the scope of the research, a combination of morphology and molecular methods has the potential to be a powerful diagnostic tool (Laha et al., 2017; Leontidou et al., 2021; Li et al., 2021; Sarwar & Takahashi, 2014). The aim of this study was to determine whether pollen can be extracted and amplified successfully after being embedded in fuchsin jelly. Signal strength and sequence quality values were used to evaluate DNA barcode amplification success. Phylogenetic analyses were used to test the reliability of the sequence data recovered.

## 2 MATERIALS AND METHODS

Plant species were collected from the summit at Gilboa Estate (MONDI Forests Ltd; 29°19′ S, 30°17′ E) in the Karkloof mountain range of the KwaZulu–Natal Midlands of South Africa.

Five plant species were gathered after the first rains of the 2021 flowering season: *Apodolirion buchanii* Baker (Amaryllidaceae); *Dimorphotheca jucunda* E. Philips (Asteraceae); *Senecio speciosus* Wiild (Asteraceae); *Hypoxis angustifolia* Lam. (Hypoxidaceae); *Moraea modesta* Killick (Iridaceae). Five flowering individuals were separately bagged and collected from each of the species. Two pollen samples were collected per individual. One sample (referred to as ‘untreated’) was used directly for DNA extractions. The second sample (‘treated’) was embedded in fuchsin jelly. Each pollen sample comprised a single anther. In the case of the Asteraceae species, an entire disc floret was removed first and then an anther removed under a dissecting microscope. The single anthers were removed using forceps and placed into individual 1.5‐ml plastic Eppendorf microcentrifuge tubes. The anther for the fuchsin‐embedded treatment was transferred to a glass slide and embedded in fuchsin jelly (~1 mm^3^, weight = 18–20 mg) by heating on a heat‐block set to 55°C for ~1 min or until just melted. With the exception of the omission of crystalline phenol, fuchsin jelly was made following (Beattie, 1971). This involves a simple procedure of mixing 150 mL glycerine with 50 g gelatine dissolved in 175 mL distilled water and then adding basic fuchsin crystals until the desired colour is attained. A cover slip was placed over the fuchsin‐embedded pollen and left for 48 h at room temperature, away from direct light.

The fuchsin‐embedded pollen was viewed at 40× magnification under a compound microscope to confirm its presence and then scraped off the glass slide and returned to the Eppendorf microcentrifuge tubes. Extraction of DNA from both pollen treatments was done using the ZYMO Quick‐DNA™ Plant/Seed Miniprep Kit (Zymo Research Group) with minor modifications suitable to the sample tissue/material: The BashingBead™ Buffer was pipetted directly into the microcentrifuge tubes containing the pollen sample, and not a ZR BashingBeadTM Lysis Tube (2.0 mm). Each pollen sample was crushed with a micropipette tip and allowed to settle for ~10 min before rinsing the pipette tip off with the buffer and removing it. The microcentrifuge tube was vortexed briefly, and the subsequent steps in the protocol were followed without amendments. Eluted DNA was stored at −20°C.

Concentrations of the eluted DNA samples were calculated by measuring the absorbance of the sample at 260/280 nm using a NanoDrop 2000 spectrophotometer (Thermo Fisher Scientific). Samples were then couriered to a commercial company (Genomyx Laboratories) for amplification and Sanger sequencing. The plant ITS region was amplified using the primer pair ITSAB101F (5′‐ACGAATTCATGGTCCGGTGAAGTGTTCG‐3′) and 26SE (5′‐TAGAATTCCCCGGTTCGCTCGCCGTTAC‐3′; Sun et al., 1994). Specimen data and DNA sequences were uploaded into BOLD under the project ‘Fuchsin‐embedded pollen’ (FFFCC; Appendix S1).

Sequences were also Blasted against NCBI (National Centre for Biotechnology Information) GenBank to authenticate and quality check the data. Forward and reverse fragments were combined to create high‐quality consensus sequences. Sequence quality was assessed from trace files using Phred quality scores (Kearse et al., 2012), sequence percentage quality (Kearse et al., 2012) and signal strength (relative fluorescence units; RFU). A Phred score measures the probability that a nucleotide has been correctly labelled. A Phred score of 30 indicates that a base has a 1 in 1000 chance of being incorrect. Phred values fall on a scale ranging from one to 60, with one being highly improbable and 60 indicating the highest accuracy possible (Ewing et al., 1998; Ewing & Green, 1998). Although not applied in all sequencing studies, a Phred score of 40 is required as the minimum threshold for human clinical samples and sequencing (Al Naiemi et al., 2006). Sequence chromatograms are categorised in Geneious Prime 2022.1 (https://www.geneious.com) by the quality scores assigned to each base (Kearse et al., 2012). Percentage quality identifies the percentage of bases in a consensus sequence which falls into each of the categories. Here sequences were binned into ‘high’, ‘medium’ and ‘low’ set at the default parameter settings. Relative fluorescence values (RFU) increase in proportion to the DNA fragment amplification success. The data were analysed with a generalised linear mixed model (GLMM) in SPSS 28 (IBM). Phred and RFU scores were analysed using models with a normal distribution and an identity link function. Plant specimen was treated as a random effect, while species, process (forward vs. reverse sequencing), treatment (fuchsin‐embedded or not) and their interactions were treated as fixed effects. For both Phred scores and RFU values, the final model was determined by the Akaike information criterion (AIC). Model degrees of freedom were adjusted using the Kenward‐Roger method (Kenward & Roger, 1997).

To determine whether fuchsin fixation led to increased levels of nucleotide misincorporation during PCR amplification, the ITS sequences were also used in phylogenetic analyses. Consensus sequences were aligned using MUSCLE (Edgar, 2004), and then the alignment was manually optimised in Bioedit (Hall et al., 2011) to ensure homology. Hypervariable sections of the final alignment that were difficult to align were removed prior to analyses. Hypervariable regions of the ITS are known to include high rates of homoplasy, which may decrease the accuracy of the reconstructed tree topologies (Barta et al., 1991, 1997; Dress et al., 2008; Ogden & Rosenberg, 2006). Maximum likelihood (ML) analyses were performed in Garli version 0.95 (Zwickl, 2006). The best‐fit model of nucleotide substitution (TIM1 + G) was selected using the AIC in JMODELTEST2 (Darriba et al., 2012). For the assessment of nodal support, 1000 bootstrap iterations were performed, and these values were annotated onto the most likely phylogeny using FIGTREE v1.3.10 (Rambaut, 2009). Average uncorrected genetic distances were calculated within and among plant species using MEGA version 11.0.10 (Tamura et al., 2011).

## 3 RESULTS

**FIGURE 1:**
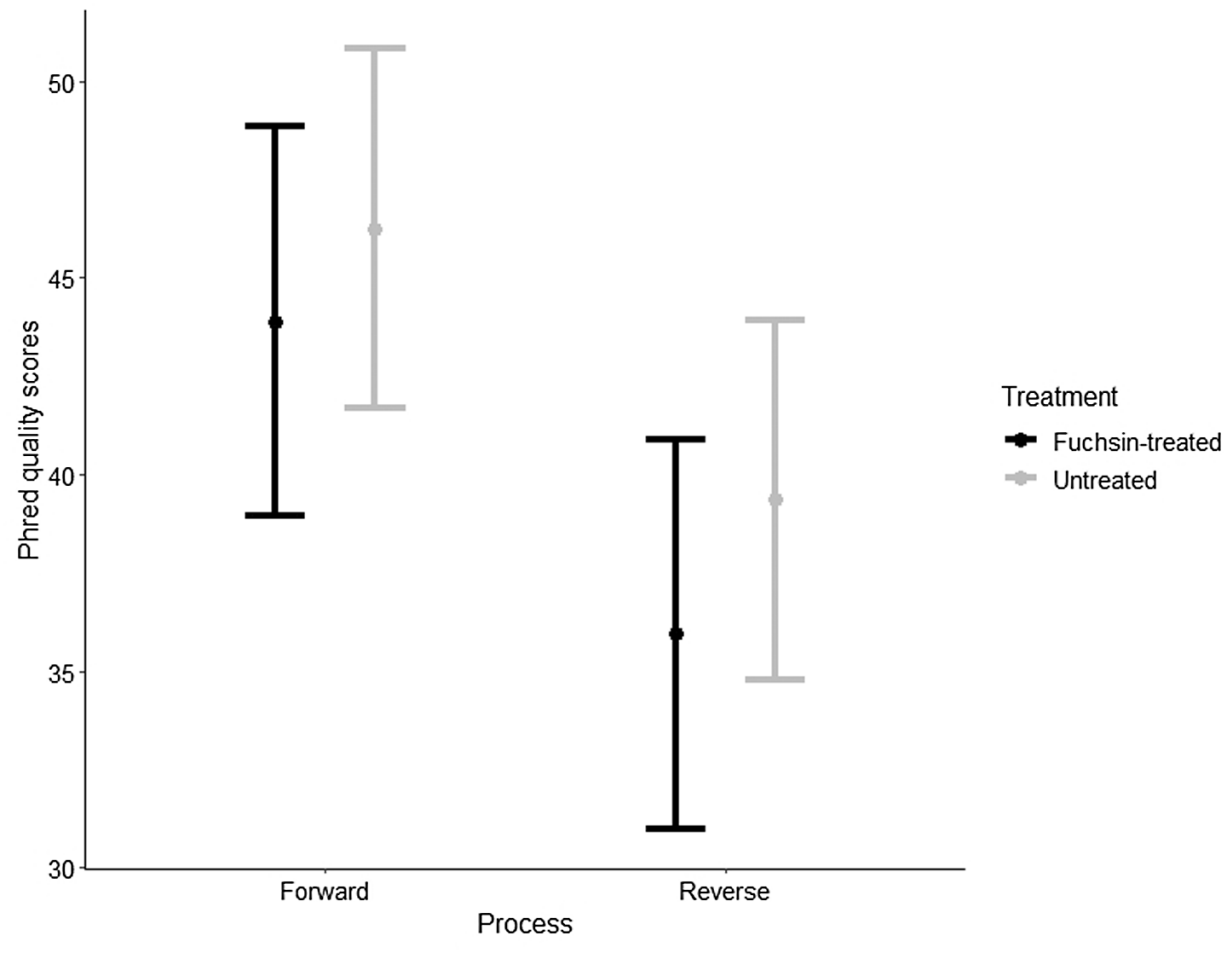
Estimated marginal means with 95% confidence intervals of Phred quality scores showing no significant difference (*p* > .05) between sequences generated using forward and reverse primers from specimens untreated and treated (fuchsin‐embedded) pollen.

**FIGURE 2:**
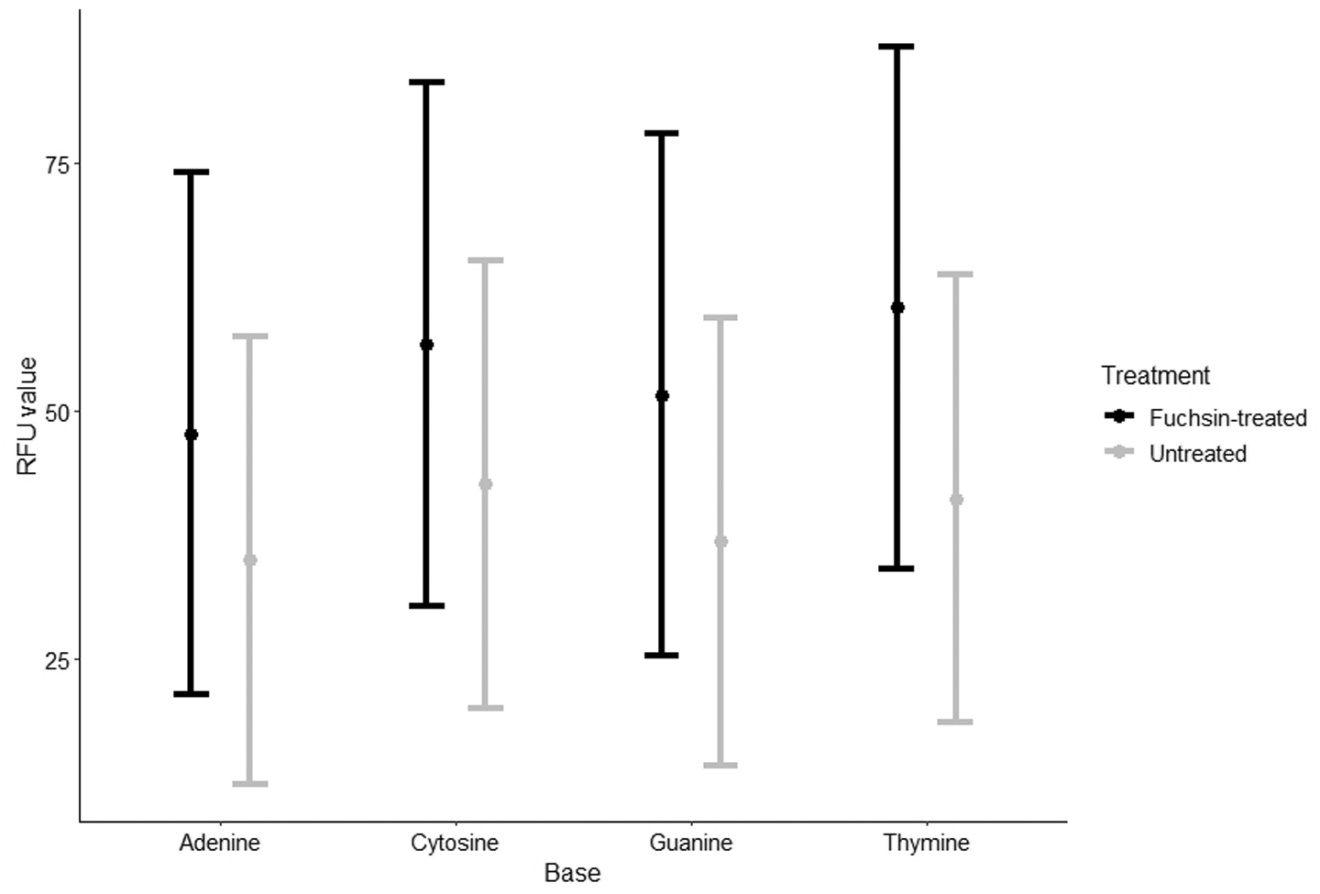
Estimated marginal means of relative fluorescence unit (RFU) values for each nucleotide for both sequences generated using forward and reverse primers with 95% confidence intervals.

**FIGURE 3:**
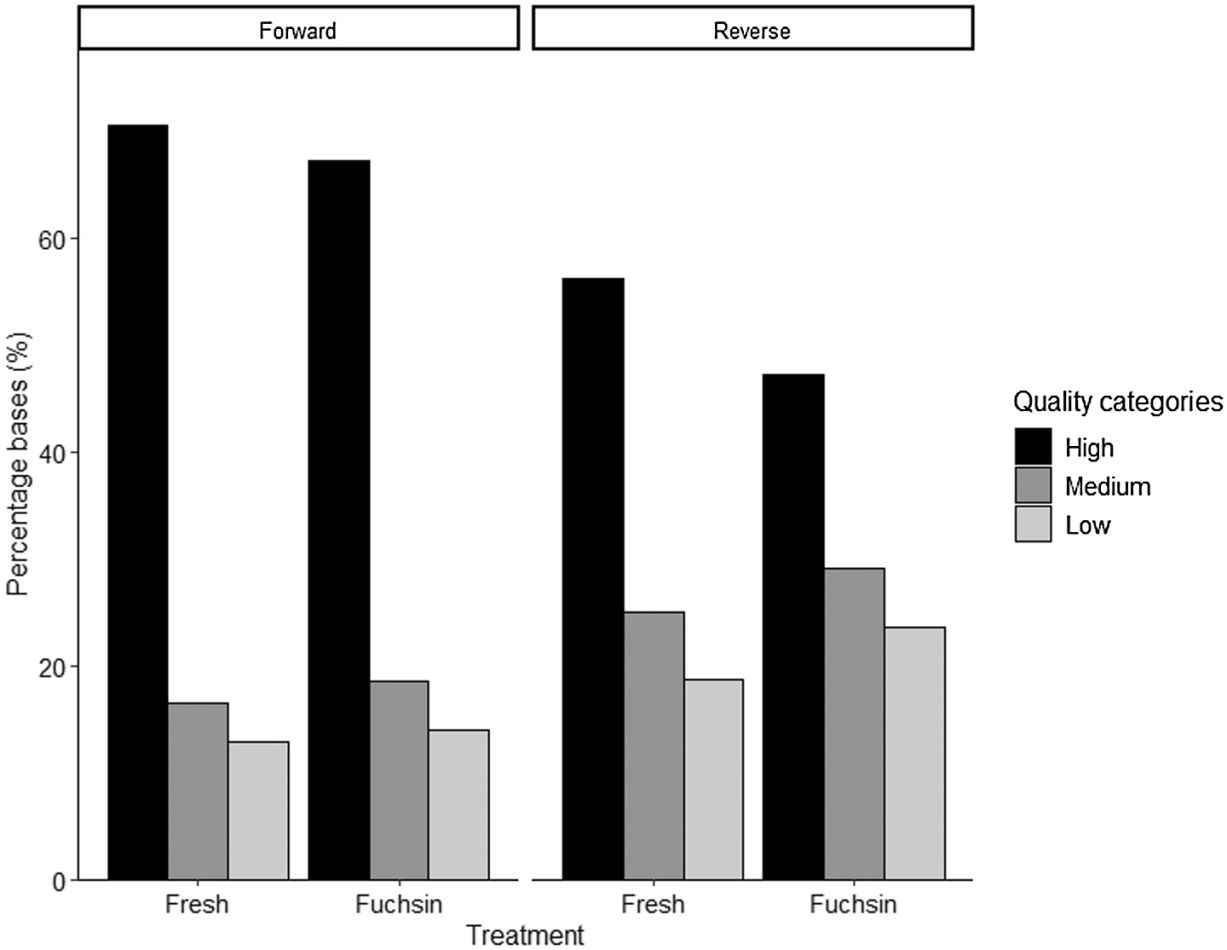
Mean percentage of base call quality, separated by category (high, medium, low) and treatment.

The best‐supported GLMM model including all possible interactions showed that fuchsin treatment had no effect on Phred scores (*F* = 1.36, *p* = .26). Extracted DNA from both treatments produced sequence trace files with high Phred quality scores (*Q*‐values, Figure 1). Overall Phred scores for untreated (*Q*‐score = 43.6, Figure 1) and fuchsin‐treated pollen DNA (*Q*‐score = 40.4, Figure 1) are beyond the minimum threshold and were not significantly different. Reverse primer amplification resulted in lower quality and a greater accumulation of erroneous base calls for both treatments across all categories (*F* = 35.6, *p* < .01, Figure 1). There was a significant effect of species on amplification success as detected by Phred scores (*F* = 5.27, *p* < .01). Likewise, RFU value was not significantly different between the treatments (*F* = 0.38, *p* = .57, Figure 2). Forward primer amplification resulted in higher RFU scores than reverse amplification (*F* = 12.25, *p* < .01). There was no effect of fuchsin on base‐call quality (Figure 3). A minimally but non‐significant improvement was found in the percentages of high‐quality base calls between the two treatments (Figure 3).

**FIGURE 4:**
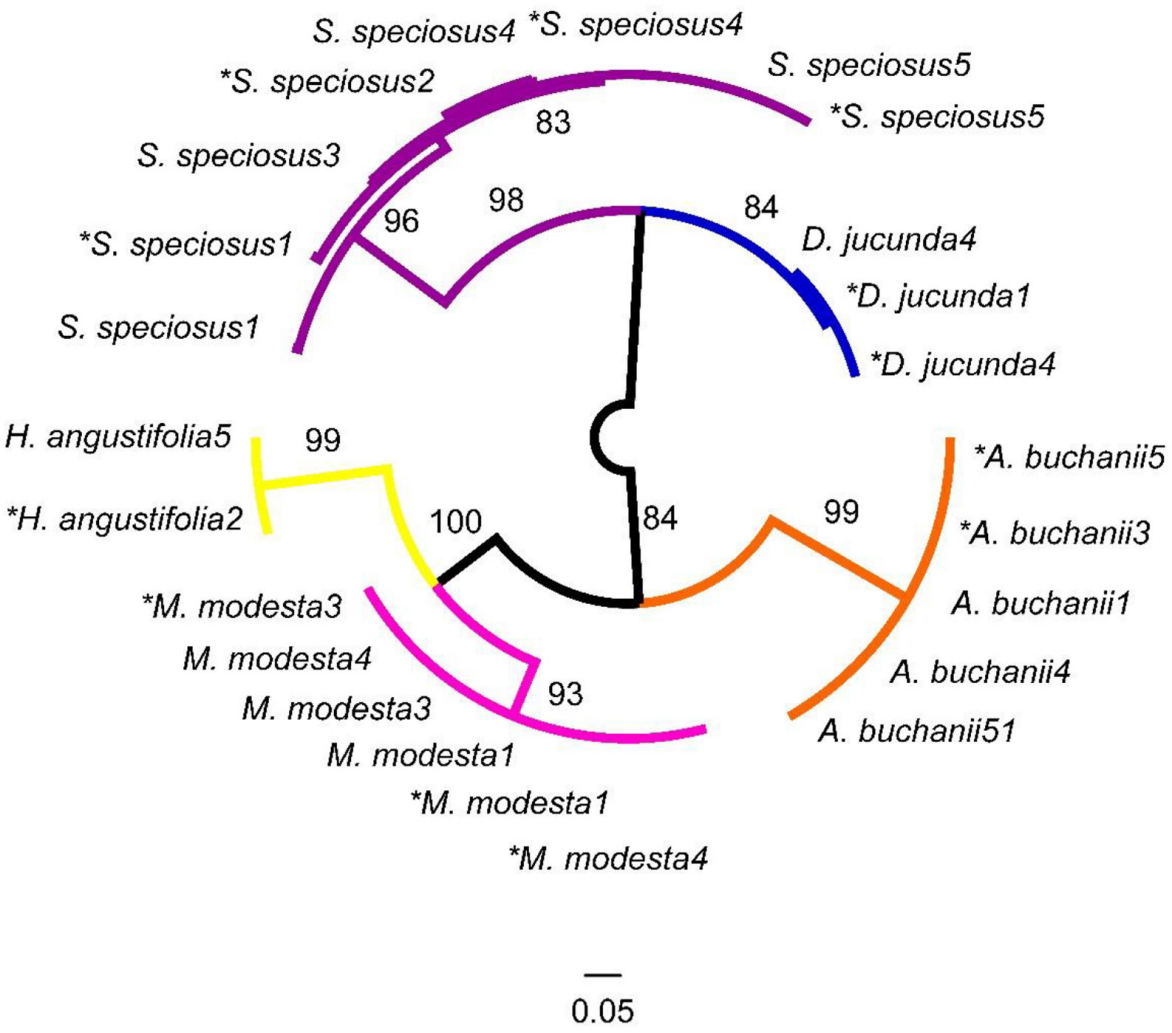
Mid‐point rooted maximum likelihood phylogeny from DNA barcode (ITS) sequences amplified from 24 individuals belonging to five species. Bootstrap values are shown on branches. Pollen DNA was extracted from untreated or after being embedded in fuchsin‐treated jelly (marked with an asterisk).

The resulting ML topology was robust. There was no shifting of the taxa, and species replicates grouped together reliably regardless of their pre‐extraction treatment, and the monophyly of species was well supported (ML bootstrap >70, Figure 4).

On average, the uncorrected genetic distances for the fuchsin‐treated among species samples ranged from 19.7% to 41.7%, which is similar to the untreated pollen genetic distances (16.5%–41.3%; Table 1). Within‐species sequence divergence was ≤1.6% (Table 1). Sequence divergence, irrespective of the effect of fuchsin, was sufficient to separate out individuals belonging to the same species.

**TABLE 1:** Uncorrected pairwise sequence distances among the species included in this study.

	*S. speciosus*	*A. Buchanii*	*D. Jacunda*	*M. modesta*	*H. angustifolia*
*S. speciosus*	**0.016**	0.431	0.192	0.339	0.325
*A. buchanii*	0.417	**0.000**	0.386	0.241	0.293
*D. jacunda*	0.197	0.376	**0.000**	0.305	0.307
*M. modesta*	0.339	0.233	0.303	**0.000**	0.165
*H. angustifolia*	0.372	0.341	0.371	0.197	**n/c**

*Note*: Fuchsin‐treated samples are below the diagonal, values from untreated pollen are above the diagonal.

Average within‐species pairwise sequence distances for fuchsin‐treated pollen are given in bold on the diagonal.

## 4 DISCUSSION

Pollen is an invaluable source of data in the fields of pollination ecology, forensics, invasive species management and others (Bell, De Vere, et al., 2016). An initial step in the process often relies on staining the unique exine structure of pollen retrieved off of target objects (e.g. pollinators, crime scene objects) in order to visualise it. However, the effect of fuchsin dye on the extraction success of pollen DNA has not yet been assessed. Here, we extracted DNA from untreated pollen and pollen which had been stained with fuchsin to determine whether pollen used previously for light microscopy could be used in downstream molecular analyses.

Extracted DNA from both untreated and fuchsin‐treated pollen produced DNA sequences of high quality, as seen by both Phred quality scores and RFU values, which did not statistically differ between the treatments. The data from both treatments also produced robust phylogenies, allowing for the reliable identification and grouping of species. This indicates that the data are accurate (Ewing et al., 1998; Ewing & Green, 1998). Increased DNA damage is associated with increased rates of nucleotide misincorporations during PCR amplification which artificially inflates sequence divergence and impacts the branching pattern seen in phylogenetic trees (Dress et al., 2008; Ogden & Rosenberg, 2006). We did not find evidence of this in the fuchsin‐treated samples. Our results indicate that DNA quality is not significantly reduced when extracted from pollen embedded on slides with fuchsin jelly, at least not over short‐term storage (up to 48 h). We did extract DNA from samples that were embedded in fuchsin for over a year (14 months). Absorbance measurement readings for these older fuchsin samples (260/280 absorbance ratio 1.04–2.07) were similar to that recovered from the samples that were left in fuchsin jelly for only 48 h (260/280 absorbance ratios between 1.6 and 1.8). We tested neither for an effect between the size of the pollen grain and the quality of amplified DNA barcodes, nor was it possible, in this study, to confirm a long‐term temporal effect of fuchsin‐embedded pollen on the recovery of DNA barcodes. These would be valuable avenues of further investigation, as both the size of the pollen grain and the duration of contact with fuchsin might contribute to diminishing the quality of recovered DNA.

## AUTHOR CONTRIBUTIONS

**Melanie B. Streicher:** Conceptualization (equal); formal analysis (equal); investigation (equal); methodology (equal); writing – original draft (equal). **Steven D. Johnson:** Conceptualization (equal); formal analysis (equal); funding acquisition (equal); supervision (equal); writing – review and editing (equal). **Sandi Willows‐Munro:** Conceptualization (equal); formal analysis (equal); project administration (equal); supervision (equal); writing – review and editing (equal).

## FUNDING INFORMATION

This work was funded by grants 46372 and 5258 to SDJ from the National Research Foundation, South Africa.

## CONFLICT OF INTEREST STATEMENT

All authors declare that they have no conflicts of interest.

## Supporting information


Appendix S1

## Data Availability

The data that support the findings of this study are openly available on BOLD Systems at https://doi.org/10.5883/DS‐FFFCC00.
