# Supplementary material for: Effect of fuchsin fixation of pollen on DNA barcode recovery
Source: Ecol Evol. 2023 Aug 31;13(9):e10475. doi: 10.1002/ece3.10475 (PMC10468989; doi:10.1002/ece3.10475)
Supplement: Supplementary file 1 — Appendix S1 [file ECE3-13-e10475-s001.docx]

| Appendix S1. Details of samples used in this study | | | | | | | | | |
| --- | --- | --- | --- | --- | --- | --- | --- | --- | --- |
|  |  |  | |  | |  |  |  |  |
| Species name | Family | Geographic locality | | Sample tissue | | Voucher type | Storage institution | Sample name | BOLD specimen number |
| *Dimorphotheca jucunda* | Asteraceae | | South Africa, KwaZulu-Natal, Mt. Gilboa | | pollen | Entire sample | NU Herbarium, University of KwaZulu-Natal | B2.1FP | FFFCC022-22 |
| *Moraea modesta* | Iridaceae | | South Africa, KwaZulu-Natal, Mt. Gilboa | | pollen | Entire sample | NU Herbarium, University of KwaZulu-Natal | A1.1RP | FFFCC001-22 |
| *Apodolirion buchananii* | Amaryllidaceae | | South Africa, KwaZulu-Natal, Mt. Gilboa | | pollen | Entire sample | NU Herbarium, University of KwaZulu-Natal | A6.5.1RP | FFFCC017-22 |
| *Senecio speciosus* | Asteraceae | | South Africa, KwaZulu-Natal, Mt. Gilboa | | pollen | Entire sample | NU Herbarium, University of KwaZulu-Natal | A3.4RP | FFFCC008-22 |
| *Senecio speciosus* | Asteraceae | | South Africa, KwaZulu-Natal, Mt. Gilboa | | pollen | Entire sample | NU Herbarium, University of KwaZulu-Natal | B3.5FP | FFFCC028-22 |
| *Apodolirion buchananii* | Amaryllidaceae | | South Africa, KwaZulu-Natal, Mt. Gilboa | | pollen | Entire sample | NU Herbarium, University of KwaZulu-Natal | B6.3FP | FFFCC031-22 |
| *Senecio speciosus* | Asteraceae | | South Africa, KwaZulu-Natal, Mt. Gilboa | | pollen | Entire sample | NU Herbarium, University of KwaZulu-Natal | A3.3RP | FFFCC007-22 |
| *Moraea modesta* | Iridaceae | | South Africa, KwaZulu-Natal, Mt. Gilboa | | pollen | Entire sample | NU Herbarium, University of KwaZulu-Natal | B1.1FP | FFFCC018-22 |
| *Moraea modesta* | Iridaceae | | South Africa, KwaZulu-Natal, Mt. Gilboa | | pollen | Entire sample | NU Herbarium, University of KwaZulu-Natal | B1.3FP | FFFCC019-22 |
| *Senecio speciosus* | Asteraceae | | South Africa, KwaZulu-Natal, Mt. Gilboa | | pollen | Entire sample | NU Herbarium, University of KwaZulu-Natal | B3.1FP | FFFCC025-22 |
| *Senecio speciosus* | Asteraceae | | South Africa, KwaZulu-Natal, Mt. Gilboa | | pollen | Entire sample | NU Herbarium, University of KwaZulu-Natal | B3.2FP | FFFCC026-22 |
| *Moraea modesta* | Iridaceae | | South Africa, KwaZulu-Natal, Mt. Gilboa | | pollen | Entire sample | NU Herbarium, University of KwaZulu-Natal | A1.4RP | FFFCC003-22 |
| *Senecio speciosus* | Asteraceae | | South Africa, KwaZulu-Natal, Mt. Gilboa | | pollen | Entire sample | NU Herbarium, University of KwaZulu-Natal | B3.4FP | FFFCC027-22 |
| *Hypoxis angustifolia* | Asparagales | | South Africa, KwaZulu-Natal, Mt. Gilboa | | pollen | Entire sample | NU Herbarium, University of KwaZulu-Natal | A4.5RP | FFFCC012-22 |
| *Moraea modesta* | Iridaceae | | South Africa, KwaZulu-Natal, Mt. Gilboa | | pollen | Entire sample | NU Herbarium, University of KwaZulu-Natal | A1.3RP | FFFCC002-22 |
| *Apodolirion buchananii* | Amaryllidaceae | | South Africa, KwaZulu-Natal, Mt. Gilboa | | pollen | Entire sample | NU Herbarium, University of KwaZulu-Natal | B6.5FP | FFFCC033-22 |
| *Senecio speciosus* | Asteraceae | | South Africa, KwaZulu-Natal, Mt. Gilboa | | pollen | Entire sample | NU Herbarium, University of KwaZulu-Natal | A3.1RP | FFFCC006-22 |
| *Apodolirion buchananii* | Amaryllidaceae | | South Africa, KwaZulu-Natal, Mt. Gilboa | | pollen | Entire sample | NU Herbarium, University of KwaZulu-Natal | A6.4RP | FFFCC015-22 |
| *Senecio speciosus* | Asteraceae | | South Africa, KwaZulu-Natal, Mt. Gilboa | | pollen | Entire sample | NU Herbarium, University of KwaZulu-Natal | A3.5RP | FFFCC009-22 |
| *Moraea modesta* | Iridaceae | | South Africa, KwaZulu-Natal, Mt. Gilboa | | pollen | Entire sample | NU Herbarium, University of KwaZulu-Natal | B1.4FP | FFFCC020-22 |
| *Dimorphotheca jucunda* | Asteraceae | | South Africa, KwaZulu-Natal, Mt. Gilboa | | pollen | Entire sample | NU Herbarium, University of KwaZulu-Natal | B2.4FP | FFFCC023-22 |
| *Dimorphotheca jucunda* | Asteraceae | | South Africa, KwaZulu-Natal, Mt. Gilboa | | pollen | Entire sample | NU Herbarium, University of KwaZulu-Natal | A2.4RP | FFFCC005-22 |
| *Hypoxis angustifolia* | Asparagales | | South Africa, KwaZulu-Natal, Mt. Gilboa | | pollen | Entire sample | NU Herbarium, University of KwaZulu-Natal | B4.2FP | FFFCC029-22 |
| *Apodolirion buchananii* | Amaryllidaceae | | South Africa, KwaZulu-Natal, Mt. Gilboa | | pollen | Entire sample | NU Herbarium, University of KwaZulu-Natal | A6.1RP | FFFCC013-22 |
